# Use of birth technology-related competence of midwives in labor management: an observational study

**DOI:** 10.1590/1980-220X-REEUSP-2024-0418en

**Published:** 2025-05-09

**Authors:** Melek Balçik Çolak, Hafize Öztürk Can

**Affiliations:** 1 Sakarya University, Faculty of Health Sciences, Department of Midwifery, Sakarya, Turkey.; 2 Ege University, Faculty of Health Sciences, Department of Midwifery, Izmir, Turkey.

**Keywords:** Midwifery, Labor, Obstetric, Observational Study, Tocologia, Trabalho de parto, Estudo Observacional

## Abstract

**Objective:**

The present study was aimed at investigating how midwives used their birth technology-related competence in labor management.

**Method:**

The study sample consisted of 14 midwives. Data were collected through interviews and by observing how the midwives followed up pregnant women. During the observations, the *“Midwife Information Form”, “Form to Assess Competence Areas of Midwives in Birth Technology”* and *“Form to Assess Technology Used in Labor”* were used. Each midwife was observed five times.

**Results:**

The assessment of the birth technology competence areas of the midwives revealed that they had deficiencies in the “caring”, “communication” sub-domains of the interpersonal skills domain and “decision making”, “traditional midwifery skills” sub-domains of the clinical competence domain.

**Conclusion:**

According to the analysis of the assessment of the technology used, the midwives working in delivery rooms tended to use low-tech devices. Traditional midwifery skills used by the participating midwives during labor were inadequate.

## RESUMO

**Objetivo:**

O objetivo do presente estudo foi investigar como as parteiras usaram sua competência relacionada à tecnologia de parto no manejo do trabalho de parto.

**Método:**

A amostra do estudo foi composta por 14 parteiras. Os dados foram coletados por meio de entrevistas e da observação de como as parteiras acompanhavam as gestantes. Durante as observações, foram utilizados o *“Formulário de Informações da Parteira”,* o *“Formulário para Avaliar as Áreas de Competência das Parteiras em Tecnologia de Parto”* e o *“Formulário para Avaliar a Tecnologia Utilizada no Trabalho de Parto”*. Cada parteira foi observada cinco vezes.

**Resultados:**

A avaliação das áreas de competência em tecnologia de parto das parteiras revelou que elas tinham deficiências nos subdomínios “cuidado” e “comunicação” do domínio de habilidades interpessoais e nos subdomínios “tomada de decisão” e “habilidades tradicionais de obstetrícia” do domínio de competência clínica.

**Conclusão:**

De acordo com a análise da avaliação da tecnologia usada, as parteiras que trabalham em salas de parto tendem a usar dispositivos de baixa tecnologia. As habilidades tradicionais de obstetrícia usadas pelas parteiras participantes durante o trabalho de parto eram inadequadas.

## INTRODUCTION

Technology makes our lives easier by increasing information sharing and shortening processes. Today, technology is developing rapidly, and it is widely used in the field of health for diagnosis, screening and treatment as it is used in all other areas of life. In this way, early diagnosis and treatment can be achieved^(1)^.

Midwives carry out maternal and child health services, women’s reproductive health services and family planning services. They also must always maintain their professionalism by following developments in science and technology. Professionalism is closely related to professional competence. Professional midwives should have clinical competence (midwifery skills) and socio-cultural skills, in finding solutions and innovations to improve, analyze, to advocate and to strengthen the well-being of women, families and communities^(2)^.

Midwives use technology while carrying out health care practices in clinical or community health services to ensure the mother’s and newborn’s health, determining risks, making clinical decisions and performing early interventions in case of emergencies. Thus, they should be competent in using technology. When technology is used, decisions should be made by analyzing information^(3)^. Technological competence should be gained based on both theoretical knowledge and experience.

In line with their clinical competencies and responsibilities, midwives should be knowledgeable about tools and machines, and their use. They should also be able to test whether they work safely, to use devices safely and effectively, to identify malfunctions, to take appropriate action, to interpret gages, and to make clinical decisions based on the interpretation of findings. While performing all these, midwives should interact with the woman and record all the processes and practices due to their legal professional obligation^(1)^. They should respect the privacy of the woman and her partner while using technology during her care, approach to her wishes and needs sensitively, and communicate with their colleagues. They should also pass the pregnant woman’s wishes to other health professionals, advocate the rights of the woman, and inform other health professionals about the care given to the woman, who should be in the center of care^(1, 4)^.

The following three typologies defined depending on midwives’ knowledge, skills and attitudes in birth technology have created a midwifery competency model: “Bureaucratic Competence (making decisions based on policies, distrusting physiological processes and traditional skills etc.)”, “Classical Professional Competence (trusting decisions made by professionals, applying traditional midwifery skills) and “New Professional Competence (considering the woman as the basis for decision making, believing in the effectiveness of physiological processes, applying traditional midwifery skills,)”^(3)^.

With the use of technology, women are now followed up with high-tech devices from the earliest period of their pregnancy to the postpartum period, and thus the risks likely to arise are intervened with technology such as (USG, continuous cardiotocography (CTG), vacuum etc.)^(5)^.

The use of technology in labor has increased the interventions made in labor, which accordingly has changed midwives’ caregiver role in labor^(3)^. However, Sinclair^(6)^ argued that midwives should also develop their basic skills in information technology and use such technology to ensure the health and safety of women, their unborn babies or newborns^(6)^. At this point, Nilsson et al.^(7)^ recommend that the midwife should communicate with the woman rather than focusing on the monitor and that they generally should us a Pinard stethoscope or Doptone device while monitoring the baby, and utilize CTG technology when necessary^(7)^. Norman^(8)^ developed clinical competence as a model based on the following five evaluable domains: clinical skill, knowledge and understanding, interpersonal characteristics, problem solving and clinical decisions, and technical skills. As Crozier et al.^(3)^ stated in their publication, they developed a Model of Birth Technology Competence, with the thought that this model would also be applicable to the assessment of midwifery competency in the use of birth technologies. Therefore, the concept of competence in birth technology has emerged as a new concept.

The number of studies in which birth technology use-related competencies of midwives are evaluated is limited. Therefore, to close this gap, the present study was conducted utilizing the Model of Birth Technology Competence published by Crozier et al.^(1, 3)^ in 2006 and 2011. This model provides evidence of a strong and transparent approach for the development, testing and validation of a theory specific to clinical midwifery practice, which contributes to the midwifery theory.

The present study was aimed at investigating birth technology use-related competencies of midwives working in the obstetrics clinic of a hospital. It was also aimed at raising midwives’ awareness of their competency areas of birth technology and the use of technology to improve the quality of care and practices by increasing the use of the “Birth Technology Competency Model” in midwifery education and practices, to provide guidance for other studies and to create a literature on this model.

## METHOD

The present study performed through interviews and observations is also a case study, in which a situation or event is examined in depth, data is collected systematically, and the event or people are examined in their real, natural environment^(9)^. Therefore, no hypothesis was put forward.

The present study was carried out with midwives working in the Obstetrics Clinic of a Public Training and Research Hospital. In the hospital, approximately 3500-4000 vaginal deliveries are performed each year. The admission of the pregnant women to the delivery room for vaginal delivery, and their follow-up are carried out by midwives. One of the midwives working in the day shift takes the anamnesis when the patient is admitted to the delivery room and conducts the first examination of the pregnant woman. Each pregnant woman is followed by a midwife in the delivery room during the labor process. The pregnant woman is examined by a specialist doctor at least once. No companions or visitors are allowed to the delivery room where the study was conducted.

### Ethical Considerations

The research was produced from the second phase of a midwifery doctoral thesis (Thesis N^o^ 606305). After the approval of the Health Sciences Institute was obtained (decision date: July 09, 2018, decision number: 88991637-302.14.01), permissions were obtained from the Scientific Research and Publication Ethics Committee (decision date: September 04, 2018, decision number: 06/10, protocol: 26) and the Chief Physician of the Training and Research Hospital (October 04, 2018, number: 24404279/702.99). The midwives included in the sample were informed about the purpose of the study, and their written consent indicating that they volunteered to participate in the study was obtained. Pregnant women were informed about the research, but no information about the pregnant women was used in the research.

### Study Population

*Participants:* All the midwives (N = 24) working in the obstetrics clinic constituted the population of the study. Of them, 14 who had been working in the delivery room for at least six months, worked only day shift, or alternating day and night shifts and volunteered to participate in the study were included in the sample. The one in charge of the clinic, those who did not always work during the day in the follow-up of pregnant women, those who worked only in the night shift, those who participated in the preliminary application of the forms and those who did not volunteer to participate in the study were not included.

*Characteristics of the Midwives Participating in the Study:* The median age of the 14 midwives included in the sample was 32 years; 11 were ≥30 years old. Other socio-demographic and professional characteristics of the participating midwives are given in Table 1.

**Table 1:** Data of Midwives on Socio-Demographical and Job-related Characteristics – Sakarya, Türkiye, 2019.

Participants (Midwives)	Age	Marital status	Education Level*	Length of service in the profession	Length of service in the delivery room	Choosing midwifery willingly	Type of Work Schedule
*M-1	25	Married	Undergraduate-Midwifery	4.5 years	7 months	Yes	Only day shift
M-2	31	Married	High school-Midwifery	6 months	6 months	Yes	Alternating day and night shifts
M-3	58	Married	High school-Midwifery	39 years	37 years	Yes	Alternating day and night shifts
M-4	33	Married	Undergraduate-Midwifery	9 years	8 months	No	Alternating day and night shifts
M-5	40	Married	Undergraduate-Midwifery	18 years	15 years	Yes	Alternating day and night shifts
M-6	31	Married	High school-Midwifery	12 years	2.5 years	Yes	Only day shift
M-7	42	Married	Undergraduate-Midwifery	18 years	13 years	No	Alternating day and night shifts
M-8	30	Married	Undergraduate-Midwifery + Teaching	7 months	7 months	Yes	Alternating day and night shifts
M-9	32	Single	Undergraduate-Midwifery	10 years	6 months	Yes	Alternating day and night shifts
M-10	30	Married	High school-Midwifery + Disaster Management + Laboratory	11 years	1 year	Yes	Alternating day and night shifts
M-11	28	Single	High school-Midwifery + Laboratorian	9 years	1 year	Yes	Alternating day and night shifts
M-12	49	Married	Associate degree-Midwifery	30 years	25 years	Yes	Alternating day and night shifts
M-13	38	Married	High school-Midwifery + Healthcare management	20 years	10 years	No	Alternating day and night shifts
M-14	27	Single	Undergraduate-Midwifery + Teaching certificate	5 years	5 years	Yes	Alternating day and night shifts

* M: Midwife. * The midwives took training other than midwifery training of their free will.

### Forms Used to Collect Data and The Process of Creating Forms

The data were collected with the following three forms prepared in line with the literature: the “Midwife Information Form”, “Form to Assess Competence Areas of Midwives in Birth Technology” and “Form to Assess Technology Used in Labor”. The forms were developed in two stages.

**The first stage**: After the Midwife Information Form was prepared, it was pilot tested and took its final form. Other forms were first translated into Turkish by the researchers, and then expert opinions were obtained to confirm the accuracy of the translation. Before the Turkish versions of the “*Form to Assess Competence Areas of Midwives in Birth Technology*” and “*Form to Assess Technology Used in Labor*” were administered to the participants in the study, they were pilot tested to find out whether the statements in them were understandable. Then opinions were obtained from six experts, one of whom was a faculty member in the Department of Sociology, one was a faculty member specialized in “Research methods” and four were faculty members in the Midwifery Department. Based on the feedback received from them, the forms were revised.

**The second stage**: In this stage, data were collected and analyzed.

### Data Collection Tools

1- *Midwife Information Form*: The form including eight items questioning the socio-demographic characteristics of the participating midwives was developed by the researchers.

2- *Form to Assess Competence Areas of Midwives in Birth Technology*: The form was developed based on the Model of Birth Technology Competence created by Crozier et al.^(1, 3)^ in their studies conducted in 2006 and 2011, to determine midwifery competency in birth technologies. According to this model, the Model of Birth Technology Competence consists of 38 applications in the following four domains and eight sub-domains: “Interpersonal Skills”, “Supporting Theoretical Knowledge”, “Professional Knowledge” and “Clinical Competence”.

Eight applications included in the “Touching” and “Caring” sub-domains of the Model of Birth Technology Competence were combined under one sub-domain: “Caring”. No changes were made in the other sub-domains. Data was collected by reducing 38 applications to 33 and the changes were based on the experts’ opinions. Then the form was divided into two parts to collect data.

**The first part**: This part was used for observational purposes during the follow-up of each pregnant woman by midwives. Each application was rated as (1) and (0) in two options: ‘Observed’ and ‘Not observed’, respectively. All the sub-domains were evaluated by observing how each midwife followed five pregnant women. If a midwife performed all the applications in all the sub-domains while following five pregnant women, she was given 165 points. One point was given to each application observed during the evaluation if the application was fulfilled.

**The second part**: In this part, data on each pregnant woman’s gestational age, cervical dilatation, fetal heart rate (FHR), the reason and time of arrival to the hospital, the mode of delivery and the duration of observation were included. These data were obtained from patient files.

3- *Form to Assess Technology Used in Labor*: The form developed based on the Model of Birth Technology Competence published by Crozier et al.^(1, 3)^ in 2006 and 2011 is used to evaluate the technology used by midwives. This form consists of the following three main categories: High-Tech Midwifery Care, Low-Tech Midwifery Care and Supportive Midwifery Care.

The technology used for the five pregnant women followed by each midwife, the applications performed according to the field of competence and category by the midwife were evaluated one by one, and a separate form was used for each pregnant woman. Each application was rated as (1) and (0) in two options: ‘Observed’ and ‘Not observed’, respectively. Findings were given based on applications fulfilled.

### Data Collection

Before data collection phase, the midwives included in the sample were interviewed, informed about the purpose of the study, and told that they would be observed by the researcher to determine “what they observed and what they did not observe” while they followed-up the pregnant women they were supposed to give care. Afterwards, the midwives who agreed to participate in the study were asked to fill in the “Midwife Information Form”, which included eight items questioning their socio- demographic characteristics.

Then the *“Form to Assess Competence Areas of Midwives in Birth Technology”* and *“Form to Assess Technology Used in Labor”* were administered to them. The main topics of the four competence areas were explained to them, the natural state of the midwives was evaluated by using the “Unattended Observation Technique” in the observations, and notes were taken. Then, data were collected using the forms.

The researcher went to the clinic at the beginning of the shift in the morning during the six-month period and was present at the shift change because operations might vary during night shifts. A midwife to be included in the study who worked in the day shift every weekday was determined by drawing lots. Blinding was achieved by not telling which midwife would be observed. Of the pregnancy follow-ups, the ones that lasted at least three hours were evaluated. To ensure objectivity, the researcher observed a midwife for a maximum of two consecutive days and continued with a different midwife on the third day. This process continued until a midwife followed up five pregnant women, and each midwife was observed at least once when following up a pregnant woman while she was giving birth.

**Figure 1:**
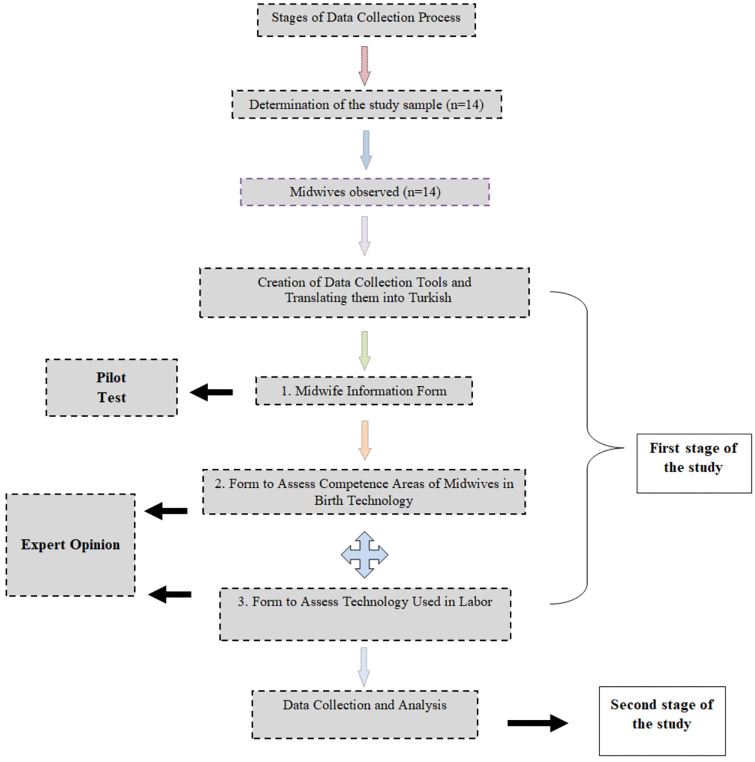
Data Collection Flowchart – Sakarya, Türkiye, 2019.

The researcher informed the pregnant women that the midwife who followed up and cared for them would be observed as part of the study, and that no information belonging to them would be used outside of the study. The data collection flow chart is shown in Figure 1. Midwives who were pilot tested were not included in the sample.

### Data Analysis

In the analysis of the data obtained from the study, the descriptive information about the midwives was given as numerical distributions. The results obtained from the Form to Assess Competence Areas of Midwives in Birth Technology and the scores corresponding to the evaluation of the competency areas of the midwives were given as a table. One point was given to each application observed during the evaluation if the application was fulfilled. Therefore, the maximum score to be obtained from all the applications was 165 points if they were all fulfilled. Applications stated in the Form to Assess Technology Used in Labor according to the competency area and category in which the technology used by the midwives during the labor were evaluated, and the observations of each midwife who followed the pregnant women were scored and given in the Excel table. Midwives were evaluated over the applications they fulfilled for five pregnant women.

## RESULTS

The findings of the present study were presented under the following two headings: the Competence Areas of Midwives in Birth Technology and the Technology Used in Birth (Table 2).

**Table 2:** Findings Regarding the Competence Areas of Midwives in Birth Technology – Sakarya, Türkiye, 2019.

Domains	Sub-domains (scores)	The number of the applications in each sub-domain	Midwives														
			M1	M2	M3	M4	M5	M6	M7	M8	M9	M10	M11	M12	M13	M14	Mean
Interpersonal Skills	Caring (20 points)	4	7	6	3	5	5	0	5	6	5	5	6	8	8	6	7.5
	Communication (20 points)	4	15	17	16	14	14	13	14	17	13	17	12	16	14	14	15
	Woman-centered care (10 points)	2	8	4	5	5	7	6	6	10	7	9	8	8	9	7	7.2
Supporting Theoretical Knowledge	Midwifery knowledge (20 points)	4	14	2	20	14	20	20	20	20	18	20	20	20	20	20	18
Professional Knowledge	Professional accountability and midwife responsibility (30 points)	6	29	21	27	21	30	30	30	30	28	30	30	30	30	30	28
Clinical Competence	Skills to use devices (30 points)	6	30	30	26	30	24	24	30	30	30	30	30	30	30	30	29.3
	Decision making (20 points)	4	16	9	16	15	16	15	15	16	15	15	15	16	15	15	15.2
	Traditional midwifery skills **(15 points)	3	4 (4V)	4 (4V)	4(3V-1F)	3 (2V-1F)	6 (4V-2F)	4 (1V- 3F)	8 (5V- 3F)	5 (4V- 1F)	6 (4V-2F)	10 (5V- 5F)	8 (1A- 4V- 3F)	11(2A- 5V- 4F)	10 (1A- 5V- 4F)	10 (1A- 5V- 4F)	6.9
Total		33	123	93	117	107	122	112	128	134	122	136	129	139	136	132	123.6

*M = Midwife

** V: Vaginal examination, F: Listening to fetal heart rate with a fetoscope, A: Abdominal examination.

During the observation, it was recorded how many times a midwife fulfilled the applications during the follow-up of five pregnant women. Because one point was given to each application observed during the evaluation if the application was fulfilled, the maximum score a midwife could obtain from all the applications was 165 points if she fulfilled all the applications. However, the scores obtained by the midwives varied between 93 and 139 points, which were below the possible maximum score, as is seen in Table 2. Especially the scores obtained from the “caring”, “woman-centered care” and “traditional midwifery skills” sub-domains by all the midwives were below the average.

2-Findings on the assessment of the technology used by midwives in labor according to their birth technology competence areas were given in Table 3. During the observation, midwives were evaluated on 3 domains, 5 sub-domains and 18 applications.

**Table 3:** Findings on Evaluating the Technology Used by Midwives in Birth According to their Birth Technology Competence Areas – Sakarya, Türkiye, 2019.

Domains	Sub-domains	Category	Applications	Midwives														
				Technology Level	1	2	3	4	5	6	7	8	9	10	11	12	13	14
Interpersonal Skills	Woman-centered care	Control of labor	Oral feeding	Supportive Midwifery Care	3	0	3	1	3	1	2	4	1	2	2	4	1	1
Supporting Theoretical Knowledge			Intravenous Hydration		3	1	1	3	1	2	1	2	1	2	3	3	0	2
	Midwifery knowledge	Neonatal Resuscitation Program (NRP)	Newborn care	High Technology	1	1	2	1	0	1	1	0	1	3	1	0	0	1
		Control of labor	Amniotomy	Low Technology	1	1	0	0	1	0	0	0	2	1	0	0	2	0
			Dosiflow (for induction)		1	1	2	3	2	2	3	2	0	5	2	2	1	1
			Prostaglandin		1	1	0	0	0	1	0	1	0	1	0	1	1	0
			Antibiotic prophylaxis	Supportive Midwifery Care	3	1	1	0	0	0	2	0	0	1	0	0	0	1
			BLADDER catheterization		0	0	2	1	2	1	1	0	1	2	1	0	1	2
			episiotomy		1	0	1	1	1	1	0	1	0	1	2	1	1	3
Professional Knowledge	Professional accountability and midwife responsibility	Bureaucracy	Computer recording	High Technology	5	5	5	5	5	5	5	5	5	5	5	5	5	5
		Follow-up of the mother and fetus	Using a partograph	Supportive Midwifery Care	4	1	1	1	1	0	0	1	2	0	1	2	0	2
Clinical Competence	Skills to use devices	Follow-up of the mother and fetus	Monitoring vital signs electronically	High Technology	0	0	0	0	1	0	0	0	0	0	0	0	2	0
			Monitoring with Cardiotocograph (CTG)		4	5	4	5	4	4	5	5	5	5	5	5	5	5
		Control of labor	Induction with Electronic Infusion Pump		2	1	0	0	0	0	0	0	1	0	0	0	0	0
		Follow-up of the mother and fetus	Monitoring Pulse-Blood pressure Manually	Low Technology	1	2	0	2	1	0	1	3	3	1	0	0	4	0
	Traditional midwifery skills	Follow-up of the mother and fetus	Hand-held Doppler (fetoscope)	Low Technology	1	0	0	1	1	2	1	1	2	3	2	4	3	3
		Alleviation of pain	Pilates	Supportive Midwifery Care	0	0	0	0	0	0	0	0	1	0	0	0	2	0
			Massage		1	0	0	0	0	0	0	0	0	0	0	0	0	0

The technology used by midwives in labor according to their birth technology competence areas are given in Table 3. Each time a midwife fulfilled these applications during five follow-ups was recorded. While all the midwives implemented high-tech CTG monitoring and computer recording in all the pregnant women they followed up, 10 midwives implemented NRP for the newborn care, but only in one of five pregnant women. They achieved low-tech applications in a small number of cases. In the supportive midwifery care sub-domain, only one midwife implemented pilates (two midwives) and massage applications, 13 monitored induction with dosiflow, 13 applied intravenous hydrations by infusion, 12 used hand Doppler, 10 used a partograph for the follow-up of the mother and fetus, 10 performed bladder catheterization, 11 performed episiotomy, and 9 monitored the pulse and blood pressure manually.

## DISCUSSION

In the present study, birth technology use-related competencies of midwives working in the obstetrics clinic were evaluated through observation and interviews. The study was conducted using the competency model in birth technology. Due to the limited number of studies in which this model was used, the comparison of the results was made using other literature related to the subject.

Competence in the technology used in childbirth also ensures the link between theory and practice. Among the competence areas of midwives working in the delivery room are the monitoring and caring of the pregnant woman they follow up. In the process starting with the admission of the pregnant woman to the clinic, her anamnesis should be taken, she should undergo physical and vaginal examination, and her risk assessment should be made. Midwives should also communicate effectively with the pregnant woman in line with their professional responsibilities and competencies, should care about the pregnant woman by making decisions about her care, and should be able to use the devices that can be used in labor fbstetrics clinic for six months. The one with the highest score was an experienced midwife who had been working in the delivery room for 30 years. Likewise, other midwives who had been working in the delivery room for many years obtained high scores. These results revealed the importance of experience in gaining competence in birth technology. The analysis of the competency areas in terms of the categories demonstrated that the lowest scores were obtained from the Interpersonal Skills domain. While the scores obtained from the “Supporting theoretical knowledge” and “professional knowledge” domains were high, the scores obtained from the “clinical competence” domain were not.

The Interpersonal Skills domain includes caring, communication, and woman-centered care sub-domains. Interpersonal Skills are very important both in the care of the pregnant woman and in team relations in labor. Interpersonal skills are also included in midwifery ethics codes of the ICM^(4)^. However, the scores they obtained from the “caring” and “woman-centered care” sub-domains were lower than was that they obtained from the communication sub-domain. In its Intrapartum Care Guidelines, WHO (2018) recommends that women’s statements and needs should be taken into account within the scope of respectful maternity care. Caring for women refers to respect for both woman’s individual rights and individualized care. It starts with putting the woman at the center of care, and ‘woman-centered care’ should be the basic philosophy in labor. The focus of the care should be ‘woman/mother’. Although the scores the midwives obtained from the “women-centered care” sub-domain were above the average, but they were not at the desired level. However, the midwives participating in the research obtained high scores from the ‘communication’ sub-domain. “Caring” together with “communication skills” play a role in the establishment of more effective interaction and cooperation between midwives, and pregnant women, mothers, and health personnel^(11, 12)^.

In addition, effective use of communication skills in taking anamnesis and taking into account the mother’s statements will ensure the detection of the risks at an early stage, and thus the pregnant woman will be followed up in a healthy way and her concerns will be relieved^(13, 14)^. Therefore, if midwives are to provide continuous support to pregnant women during labor, they should have good communication skills and provide respectful care to pregnant women. Midwives’ establishing effective communication is the main factor for the individuality of the birth and it will ensure the pregnant woman’s active participation in the process and will encourage her during labor. Thus, the use of analgesia and anesthesia is reduced, the rate of interventions is reduced, the pregnant woman’s self-confidence and sense of success increase, her active participation in birth is ensured, the duration of the labor is shortened, a safe delivery environment is created with the continuous support of midwives, mother-baby bonding is strengthened, effective breastfeeding is ensured, adaptation to the postpartum period is facilitated and the pregnant woman’s comfort and maternal satisfaction at birth increases^(12, 13, 15)^.

It was observed that midwives during the daytime working hours could not spare enough time for pregnant women, which was probably because the level of bureaucratic work during the daytime working hours in the clinic was high, the number of team members working in the clinic was high, and each team member had some expectations from midwives.

In the present study, it was noticed that all the midwives carried out the category of ‘bureaucracy’ in the “professional knowledge” sub-domain for all the pregnant women. In this category, the midwives exhibited the elements of Bureaucratic Competence in applications. Among the reasons why bureaucracy is more dominant are legal litigation concerns, the importance given to protocols and guidelines, and the inclusion of physicians in responsibility^(3)^. The analysis of the results demonstrated that all the midwives performed “computer recording” application in the “bureaucracy” category. This proves that although the midwives were aware of their professional responsibilities and accountability, they also attached importance to recording indicating that they worked as ‘medical secretaries’.

The midwives’ inadequacy observed in the behavior of caring for women was probably due to the differences between their ages, lengths of service in the profession and in the delivery room, and education levels. Two other domains of birth technology competency are *Supporting Theoretical Knowledge* (midwifery knowledge) and *Professional Knowledge* (professional accountability and midwife responsibility). The scores the midwives obtained from these domains of birth technology competency were high.

The midwives carried out “newborn care” application in the “Midwifery Knowledge” sub-domain using high technology. In the present study, it was observed that the midwives implemented the applications in the second phase of labor at least once, but unfortunately, not all the midwives. Of the 14 midwives, ten used high technology while performing newborn care because they had previously used that technology at least once. Applications such as amniotomy, dosiflow induction and prostaglandin, which are in the “Control of labor” category of midwifery knowledge subdomain were performed using low technology. As is seen in Table 3, the midwives did not fulfill the applications such as antibiotic use, bladder catheterization and episiotomy included in the Control of Labor category in all the pregnant women. Implementation of these applications at a low rate is considered as a positive approach because the midwifery knowledge mentioned here means intervention in labor. Because the hospital where the present study was conducted is a tertiary one, decisions on induction application and antibiotic prophylaxis are made by physicians. Midwives are only responsible for performing the applications and following the process. In addition, they should inform the physicians about complications that arise or are likely to arise. In their study conducted with primiparous pregnant women, Balcik Colak and Ozturk Can Can^(19, 20)^ stated that 88% of the pregnant women underwent bladder catheterization during delivery, which was consistent with our findings. Since interventions in labor are performed at a high rate in the world, WHO does not recommend intervention in labor unless necessary^(12)^.

In the Clinical Competence domain, all the midwives obtained high scores from the “Skills to Use Devices” sub-domain. However, the score they obtained from the “Traditional Midwifery Skills” sub-domain was below average. In addition, it was observed that only one midwife performed massage application. Although 12 midwives monitored the FHR with a fetoscope, which is in the same category, they did not carry it out in all the pregnant women they followed up. Instead, they preferred CTG. The WHO does not recommend continuous vaginal examination during labor. In the present study, the reason why the midwives did not perform adequate abdominal examination was probably because physicians were the ones to decide on the procedures to be undergone by the pregnant women. That is why they performed CTG and USG.

In addition, the midwives did not monitor vital signs electronically in the category of monitoring the mother and fetus, but all the midwives used CTG. These two applications are carried out by using advanced technology. However, this indicates that midwives did not monitor contractions by touching the pregnant woman with their hands. Since the follow-up of the pregnant woman with CTG also made it possible to perform evaluations in a shorter time, the midwives did not maintain long-term contact with the pregnant woman. Because midwives spend short time with the pregnant woman, they cannot listen to the pregnant woman adequately and thus cannot perform appropriate interventions to relieve the woman’s pain. As a routine practice, CTG is important since it is used to determine and detect fetal risks early and to prevent complications^(21)^. The WHO, American College of Nurse Midwives, and the National Institute for Health and Care Excellence (NICE) in the United Kingdom, all of which deal with maternal and child health, do not recommend the routine use of CTG but recommend intermittent auscultation for fetal monitoring except in risky situations^(12, 22, 23)^. In some studies, in which the opinions of health professionals about fetal monitoring in labor were included, it was reported that health professionals felt safer with CTG and wanted to use it due to legal concerns^(24)^.

Among the technological applications used in the follow-up of labor, monitoring vital signs electronically, and induction with an electronic infusion pump are considered high-tech applications whereas manual monitoring of vital signs is stated as a low-tech application^(3)^. Only two of the midwives participating in the present study monitored vital signs electronically, because the other midwives stated that they did not trust digital measurements in the measurement of vital signs. Therefore, most of the midwives used the low-tech manual system to monitor heart rate and blood pressure.

Induction applications with electronic infusion pump was performed by only three of them. They actively used low technology in induction applications because they had problems in using the induction pump and lacked knowledge about how to use it, which suggests that they need training on the use of devices and technology.

It is reported that the evaluation of the fetal head with routine USG is not beneficial in low-risk pregnancies and that it is associated with higher cesarean rates^(25)^. In some observational studies, it is reported that repetitive USG gives more successful results than does manual clinical examination in the diagnosis of labor that does not progress in the first and second stages of labor^(26)^. The use of technological methods such as CTG and USG has a very important place in determining risky situations. However, their routine use causes a decrease in touching the pregnant woman and reduces interaction with the pregnant woman. Touching has been reported to have positive physiological and psychological effects in relieving birth pain, to reduce anxiety and pain scores significantly and to shorten the duration of labor^(27, 28)^. In addition, if midwives are aware of their areas of competence and autonomy, they will be able to make the right decision about the follow-up of pregnant women admitted to the obstetrics clinic, to provide the necessary information and guidance, and to ensure the maximum benefit for the health of the mother and baby.

Since the use of technological devices serves the main purpose, the use of technology in labor becomes the main necessity. It should be noted that using devices is not a goal but a tool, and awareness about their intended use should be raised^(30)^. In their studies, Cowie and Floyd^(29)^ expressed their concerns that midwives were terminating the real functions of midwifery due to the technological devices they used in midwifery practices. Consistent with the results of the present study, data obtained during the interviews held with the midwives participating in Cowie and Floyd’s study indicated that the use of technology decreased the touch on the pregnant, and that manual skills and practices in midwifery were put into the background.

The scores the participating midwives obtained from the “Decision Making” sub-domain of the “Clinical Competence” domain were above the average, but not at a high level. In the present study, the clinical decision-making levels of the participants were not evaluated separately. However, it is thought that the decision-making power and autonomy of the midwives should be further strengthened. This will also promote female-centered care^(19)^. It is thought that the midwife who obtained the lowest score from the “Decision Making” subdomain did not yet gain competence in decision making because her length of service in the delivery room was short and because her midwifery education was at the high school level.

The midwives’ approach should be based on holistic care. The focus should be on the individualized use of technology. The routine use of technology should be limited, because it leads to a decline in the provision of individualized care, and negatively affects the process of being with women. It is important to analyze the effects of technology use in the evaluation of intrapartum follow-up and results on the role of midwives and on the woman’s birth experience^(30)^. In the technocratic model, it is thought that the work of health professionals is facilitated^(2, 6)^.

According to the results of the present study, midwives advocated new professional competency typologies in the interview phase, but they applied the bureaucratic competency typology. According to the results of the interviews held with the midwives in Crozier et al.’s^(3)^ study, although advocating Professional Competence, they exhibited the characteristics of Bureaucratic Competence in practice.

The fact that participating midwives’ education levels and length of service in the profession varied and that some of them received training other than midwifery education may have affected their competency. In addition, the physical conditions of the delivery area, work-centered working conditions, inadequate delivery area environment, an inadequate number of personnel and inadequate in-service training may also have affected the results of the present study. However, these thoughts should only be considered as assumptions, since the main purpose in the present study was not to determine the factors that affected competencies related to birth technologies. In addition to the use of technology, traditional midwifery practices such as being with the patient, listening, counseling and empathy should also be included in order not to stray away from midwife-centered care.

## CONCLUSION

In the present observational study conducted to investigate midwives’ competency areas and technology use during labor, it was observed that the participating midwives had some inadequacies regarding the use of technology in labor and in areas such as interpersonal skills and clinical competence. The results obtained based on our observations are as follows:

- In general, the participating midwives working in the delivery room used low-tech devices.
- Traditional midwifery skills used by the participating midwives during labor were inadequate.

In line with these results, to improve midwives’ competency areas and to eliminate their inadequacies in the use of technology in labor, it is recommended that:

- In midwifery education, midwives should be given technology education on how to use technological devices, and how to correctly interpret and intervene in the recording outputs of the devices, and that their technological competence needs should be met, supported and improved with training.
- While providing counseling, midwives should inform women about the use of technology to reduce its negative effects on the health of pregnant women, mothers and babies, that they should ensure that necessary precautions are taken in the use of devices, and that they should fulfill their advocacy roles in training and counseling.
- Policy makers should update data on the use of technology in midwifery education programs and that the authority of midwives be expanded by policy makers regarding the use of technological devices.
