# Supplementary material for: Use of birth technology-related competence of midwives in labor management: an observational study
Source: Rev Esc Enferm USP. 2025 May 9;59:e20240418. doi: 10.1590/1980-220X-REEUSP-2024-0418en (PMC12064084; doi:10.1590/1980-220X-REEUSP-2024-0418en)
Supplement: Translation

# Translation: Uso de la tecnología del parto relacionada con la competencia de las matronas en la gestión del parto: un estudio observacional

**DOI:** 10.1590/1980-220X-REEUSP-2024-0418es

**Authors:** Melek Balçik Çolak, Hafize Öztürk Can

**Affiliations:** 1 Sakarya University, Faculty of Health Sciences, Department of Midwifery, Sakarya, Turkey.; 2 Ege University, Faculty of Health Sciences, Department of Midwifery, Izmir, Turkey.

**Keywords:** Partería, Trabajo de Parto, Estudio Observacional

## Abstract

**Objetivo:**

El presente estudio tenía como objetivo investigar cómo utilizaban las matronas su competencia relacionada con la tecnología del parto en la gestión del parto.

**Método:**

La muestra del estudio estaba formada por 14 matronas. Los datos se recogieron mediante entrevistas y observando cómo las matronas realizaban el seguimiento de las embarazadas. Durante las observaciones se utilizaron el *“Formulario de información para matronas”,* el *“Formulario para evaluar las áreas de competencia de las matronas en tecnología del parto”* y el *“Formulario para evaluar la tecnología utilizada en el parto”.* Cada matrona fue observada cinco veces.

**Resultados:**

La evaluación de las áreas de competencia en tecnología del parto de las matronas reveló que presentaban deficiencias en los subdominios «cuidado», «comunicación» del dominio de habilidades interpersonales y «toma de decisiones», «habilidades tradicionales de partería» del dominio de competencia clínica.

**Conclusiones:**

Según el análisis de la evaluación de la tecnología utilizada, las matronas que trabajan en salas de partos tendían a utilizar dispositivos de baja tecnología. Las habilidades tradicionales de partería utilizadas por las matronas participantes durante el parto eran inadecuadas.

## INTRODUCCIÓN

La tecnología nos facilita la vida al aumentar el intercambio de información y acortar los procesos. Hoy en día, la tecnología se está desarrollando rápidamente y se utiliza ampliamente en el campo de la salud para el diagnóstico, la detección y el tratamiento, al igual que en todas las demás áreas de la vida. De esta manera, se puede lograr un diagnóstico y tratamiento tempranos^(1)^. Las matronas prestan servicios de salud maternoinfantil, servicios de salud reproductiva de la mujer y servicios de planificación familiar. También deben mantener siempre su profesionalidad siguiendo los avances de la ciencia y la tecnología. La profesionalidad está estrechamente relacionada con la competencia profesional. Las matronas profesionales deben tener competencia clínica (habilidades de partería) y habilidades socioculturales, para encontrar soluciones e innovaciones para mejorar, analizar, defender y fortalecer el bienestar de las mujeres, las familias y las comunidades^(2)^. Las matronas utilizan la tecnología en la práctica de la atención sanitaria en los servicios de salud clínicos o comunitarios para garantizar la salud de la madre y del recién nacido, determinar los riesgos, tomar decisiones clínicas y realizar intervenciones tempranas en caso de emergencia. Por lo tanto, deben ser competentes en el uso de la tecnología. Cuando se utiliza la tecnología, las decisiones deben tomarse analizando la información^(3)^. La competencia tecnológica debe adquirirse a partir de los conocimientos teóricos y la experiencia.

En consonancia con sus competencias y responsabilidades clínicas, las matronas deben conocer las herramientas y máquinas, así como su uso. También deben ser capaces de comprobar si funcionan de forma segura, utilizar los dispositivos de forma segura y eficaz, identificar averías, tomar las medidas adecuadas, interpretar los indicadores y tomar decisiones clínicas basadas en la interpretación de los resultados. Mientras realizan todas estas tareas, las matronas deben interactuar con la mujer y registrar todos los procesos y prácticas debido a su obligación profesional legal^(1)^. Deben respetar la privacidad de la mujer y su pareja cuando utilicen tecnología durante su atención, abordar sus deseos y necesidades con sensibilidad y comunicarse con sus colegas. También deben transmitir los deseos de la mujer embarazada a otros profesionales de la salud, defender los derechos de la mujer e informar a otros profesionales de la salud sobre la atención prestada a la mujer, que debe ser el centro de la atención^(1, 4)^.

Las tres tipologías siguientes, definidas en función de los conocimientos, habilidades y actitudes de las matronas en la tecnología del parto, han creado un modelo de competencia de partería: «Competencia burocrática (tomar decisiones basadas en políticas, desconfiar de los procesos fisiológicos y las habilidades tradicionales, etc.)», «Competencia profesional clásica (confiar en las decisiones tomadas por los profesionales, aplicar las habilidades tradicionales de partería) y «Nueva competencia profesional (considerar a la mujer como la base para la toma de decisiones, creer en la eficacia de los procesos fisiológicos, aplicar las habilidades tradicionales de partería)»^(3)^.

Con el uso de la tecnología, ahora se realiza un seguimiento de las mujeres con dispositivos de alta tecnología desde el primer período de su embarazo hasta el posparto, y así se interviene en los riesgos que puedan surgir con tecnología como la ecografía, la cardiotocografía continua (CTG), el vacío (ventosa), etc^(5)^.

El uso de la tecnología en el parto ha aumentado las intervenciones realizadas en el parto, lo que, en consecuencia, ha cambiado el papel de las matronas como cuidadoras en el parto^(3)^. Sin embargo, Sinclair^(6)^ argumentó que las matronas también deberían desarrollar sus habilidades básicas en tecnología de la información y utilizar dicha tecnología para garantizar la salud y la seguridad de las mujeres, sus bebés nonatos o recién nacidos. En este punto, Nilsson et al.^(7)^ recomiendan que la matrona se comunique con la mujer en lugar de concentrarse en el monitor y que, por lo general, utilice un estetoscopio Pinard o un dispositivo Doptone mientras monitorea al bebé, y que utilice la tecnología CTG cuando sea necesario. Norman^(8)^ desarrolló la competencia clínica como un modelo basado en los siguientes cinco dominios evaluables: habilidad clínica, conocimiento y comprensión, características interpersonales, resolución de problemas y decisiones clínicas, y habilidades técnicas. Como Crozier et al.^(3)^ afirmaron en su publicación, desarrollaron un Modelo de Competencia en Tecnología del Parto, con la idea de que este modelo también sería aplicable a la evaluación de la competencia de las matronas en el uso de tecnologías del parto. Por lo tanto, el concepto de competencia en tecnología del parto ha surgido como un nuevo concepto.

El número de estudios en los que se evalúan las competencias de las matronas relacionadas con el uso de la tecnología del parto es limitado. Por lo tanto, para cerrar esta brecha, el presente estudio se llevó a cabo utilizando el Modelo de Competencia en Tecnología del Parto publicado por Crozier et al.^(1, 3)^ en 2006 y 2011. Este modelo proporciona evidencia de un enfoque sólido y transparente para el desarrollo, prueba y validación de una teoría específica para la práctica clínica de partería, que contribuye a la teoría de la partería.

El presente estudio tenía como objetivo investigar las competencias relacionadas con el uso de la tecnología del parto de las matronas que trabajan en la clínica de obstetricia de un hospital. También tenía como objetivo concienciar a las matronas sobre sus áreas de competencia en tecnología del parto y el uso de la tecnología para mejorar la calidad de la atención y las prácticas mediante el aumento del uso del «Modelo de competencias en tecnología del parto» en la educación y las prácticas de partería, proporcionar orientación para otros estudios y crear una bibliografía sobre este modelo.

## MÉTODO

El presente estudio, realizado a través de entrevistas y observaciones, es también un estudio de caso, en el que se examina en profundidad una situación o un acontecimiento, se recogen datos de forma sistemática y se examina el acontecimiento o a las personas en su entorno real y natural^(9)^. Por lo tanto, no se planteó ninguna hipótesis.

El presente estudio se llevó a cabo con matronas que trabajan en la Clínica de Obstetricia de un Hospital Público de Formación e Investigación. En el hospital se realizan aproximadamente entre 3500 y 4000 partos vaginales cada año. Las matronas se encargan de la admisión de las mujeres embarazadas en la sala de partos para el parto vaginal y de su seguimiento. Una de las matronas que trabaja en el turno de día toma la anamnesis cuando la paciente ingresa en la sala de partos y realiza el primer examen de la embarazada. Cada embarazada es seguida por una matrona en la sala de partos durante el proceso de parto. La embarazada es examinada por un médico especialista al menos una vez. No se permite la entrada de acompañantes ni visitantes en la sala de partos donde se realizó el estudio.

### Consideraciones Éticas

La investigación se realizó a partir de la segunda fase de una tesis doctoral de partería (tesis n.º 606305). Tras obtener la aprobación del Instituto de Ciencias de la Salud (fecha de la decisión: 9 de julio de 2018, número de la decisión: 88991637-302.14.01), se obtuvieron permisos del Comité de Ética de Investigación Científica y Publicaciones (fecha de la decisión: 4 de septiembre de 2018, número de la decisión: 06/10, protocolo: 26) y del médico jefe del Hospital de Formación e Investigación (4 de octubre de 2018, número: 24404279/702.99). Se informó a las matronas incluidas en la muestra sobre el propósito del estudio y se obtuvo su consentimiento por escrito indicando que se ofrecían voluntarias para participar en el estudio. Se informó a las mujeres embarazadas sobre la investigación, pero no se utilizó ninguna información sobre las mujeres embarazadas en la investigación.

### Población del Estudio

*Participantes:* Todas las matronas (N = 24) que trabajaban en la clínica de obstetricia constituían la población del estudio. De ellas, se incluyeron en la muestra 14 que habían estado trabajando en la sala de partos durante al menos seis meses, que trabajaban solo en el turno de día o que alternaban turnos de día y de noche y se ofrecieron voluntarias para participar en el estudio. No se incluyeron las encargadas de la clínica, las que no siempre trabajaban durante el día en el seguimiento de las mujeres embarazadas, las que trabajaban solo en el turno de noche, las que participaron en la aplicación preliminar de los formularios y las que no se ofrecieron como voluntarias para participar en el estudio.

*Características de las matronas que participaron en el estudio:* la edad media de las 14 matronas incluidas en la muestra fue de 32 años; 11 tenían ≥30 años. En la Tabla 1 se muestran otras características sociodemográficas y profesionales de las matronas participantes.

**Tabla 1:** Datos de las matronas sobre características sociodemográficas y laborales – Sakarya, Turquía, 2019.

Participantes (Matronas)	Edad	Estado civil	Nivel de estudios*	Antigüedad en la profesión	Duración del servicio en la sala de partos	Eligió voluntariamente la partería	Tipo de horario de trabajo
*M-1	25	Casada	Licenciatura en obstetricia	4,5 años	7 meses	Sí	Sólo turno de día
M-2	31	Casada	Bachillerato-Medicina	6 meses	6 meses	Sí	Turnos alternos de día y de noche
M-3	58	Casada	Bachillerato-Matrona	39 años	37 años	Sí	Turnos alternos de día y de noche
M-4	33	Casada	Licenciatura-Matrona	9 años	8 meses	No	Turnos alternos de día y de noche
M-5	40	Casada	Licenciatura-Matrona	18 años	15 años	Sí	Turnos alternos de día y de noche
M-6	31	Casada	Bachillerato-Matrona	12 años	2,5 años	Sí	Sólo turno de día
M-7	42	Casada	Licenciatura-Matrona	18 años	13 años	No	Turnos alternos de día y de noche
M-8	30	Casada	Licenciatura-Matrona + Enseñanza	7 meses	7 meses	Sí	Turnos alternos de día y de noche
M-9	32	Únic	Licenciatura-Matrona	10 años	6 meses	Sí	Turnos alternos de día y de noche
M-10	30	Casada	Bachillerato-Matrona + Gestión de catástrofes + Laboratorio	11 años	1 año	Sí	Turnos alternos de día y de noche
M-11	28	Únic	Bachillerato-Matrona + Laboratorista	9 años	1 año	Sí	Turnos alternos de día y de noche
M-12	49	Casada	Diplomado en obstetricia	30 años	25 años	Sí	Turnos alternos de día y de noche
M-13	38	Casada	Bachillerato-Matrona + Gestión sanitaria	20 años	10 años	No	Turnos alternos de día y de noche
M-14	27	Únic	Licenciatura-Matrona + Certificado de enseñanza	5 años	5 años	Sí	Turnos alternos de día y de noche

* M: Matrona. *Las matronas siguieron una formación distinta a la de partería por voluntad propia.

#### Formularios Utilizados Para Recolectar Datos y el Proceso de Creación de Formularios

Los datos se recopilaron con los tres formularios siguientes, preparados de acuerdo con la bibliografía: el «Formulario de información de la matrona», el «Formulario para evaluar las áreas de competencia de las matronas en la tecnología del parto» y el «Formulario para evaluar la tecnología utilizada en el trabajo de parto». Los formularios se desarrollaron en dos etapas.

**Primera etapa**: Después de preparar el Formulario de información de la matrona, se realizó una prueba piloto y se le dio su forma final. Los investigadores tradujeron primero otros formularios al turco y, a continuación, se obtuvieron opiniones de expertos para confirmar la exactitud de la traducción. Antes de que las versiones turcas del «*Formulario para evaluar las áreas de competencia de las matronas en la tecnología del* parto» y el «*Formulario para evaluar la tecnología utilizada en el* parto» se administraran a los participantes en el estudio, se realizaron pruebas piloto para averiguar si las afirmaciones que contenían eran comprensibles. Luego se obtuvieron las opiniones de seis expertos, uno de los cuales era miembro del cuerpo docente del Departamento de Sociología, uno era miembro del cuerpo docente especializado en «Métodos de investigación» y cuatro eran miembros del cuerpo docente del Departamento de Partería. Sobre la base de los comentarios recibidos de ellos, se revisaron los formularios.

**Segunda etapa**: En esta etapa, se recolectaron y analizaron los datos.

### Herramientas de Recolección de Datos

1- *Formulario de información de la matrona*: Los investigadores desarrollaron el formulario, que incluye ocho preguntas sobre las características sociodemográficas de las matronas participantes.

2- *Formulario para evaluar las áreas de competencia de las matronas en la tecnología del* parto: El formulario se desarrolló a partir del Modelo de Competencia en Tecnología del Parto creado por Crozier et al.^(1, 3)^ en sus estudios realizados en 2006 y 2011, para determinar la competencia de las matronas en las tecnologías del parto. Según este modelo, el Modelo de Competencia en Tecnologías del Parto consta de 38 aplicaciones en los siguientes cuatro dominios y ocho subdominios: «Habilidades interpersonales», «Soporte de conocimientos teóricos», «Conocimientos profesionales» y «Competencia clínica».

Ocho aplicaciones incluidas en los subdominios «Tocar» y «Cuidar» del Modelo de Competencia en Tecnologías del Parto se combinaron en un subdominio: «Cuidar». No se realizaron cambios en los otros subdominios. Los datos se recolectaron reduciendo 38 aplicaciones a 33 y los cambios se basaron en las opiniones de los expertos. Luego, el formulario se dividió en dos partes para recolectar datos.

**La primera parte**: Esta parte se utilizó con fines de observación durante el seguimiento de cada mujer embarazada por parte de las matronas. Cada aplicación se calificó como (1) y (0) en dos opciones: «Observado» y «No observado», respectivamente. Todos los subdominios se evaluaron observando cómo cada matrona seguía a cinco mujeres embarazadas. Si una matrona realizaba todas las aplicaciones en todos los subdominios mientras seguía a cinco mujeres embarazadas, se le otorgaban 165 puntos. Se otorgaba un punto a cada aplicación observada durante la evaluación si la aplicación se cumplía.

**La segunda parte**: En esta parte, se incluyeron datos sobre la edad gestacional de cada mujer embarazada, la dilatación cervical, la frecuencia cardíaca fetal (FCF), el motivo y la hora de llegada al hospital, el modo de parto y la duración de la observación. Estos datos se obtuvieron de los expedientes de las pacientes.

3- *Formulario para evaluar la tecnología utilizada en el parto*: El formulario desarrollado a partir del Modelo de Competencia Tecnológica en el Parto publicado por Crozier et al.^(1, 3)^ en 2006 y 2011 se utiliza para evaluar la tecnología utilizada por las matronas. Este formulario consta de las tres categorías principales siguientes: Atención obstétrica de alta tecnología, atención obstétrica de baja tecnología y atención obstétrica de apoyo.

La tecnología utilizada para las cinco mujeres embarazadas seguidas por cada matrona, las aplicaciones realizadas según el campo de competencia y la categoría por la matrona se evaluaron una por una, y se utilizó un formulario separado para cada mujer embarazada. Cada aplicación se calificó como (1) y (0) en dos opciones: «Observado» y «No observado», respectivamente. Los resultados se dieron en función de las aplicaciones cumplidas.

### Recolección de Datos

Antes de la fase de recolección de datos, se entrevistó a las matronas incluidas en la muestra, se les informó sobre el propósito del estudio y se les dijo que serían observadas por el investigador para determinar «lo que observaron y lo que no observaron» mientras realizaban el seguimiento de las mujeres embarazadas a las que se suponía que debían atender. Posteriormente, se pidió a las matronas que aceptaron participar en el estudio que rellenaran el «Formulario de información de la matrona», que incluía ocho preguntas sobre sus características sociodemográficas.

Luego se les administró el «Formulario para evaluar las áreas de competencia de las matronas en la tecnología del parto» y el «Formulario para evaluar la tecnología utilizada en el trabajo de parto». Se les explicaron los temas principales de las cuatro áreas de competencia, se evaluó el estado natural de las matronas utilizando la «Técnica de observación sin supervisión» en las observaciones y se tomaron notas. Luego, se recolectaron datos utilizando los formularios.

El investigador acudió a la clínica al comienzo del turno de la mañana durante el período de seis meses y estuvo presente en el cambio de turno porque las operaciones podrían variar durante los turnos de noche. Se determinó por sorteo qué matrona se incluiría en el estudio y trabajaría en el turno de día todos los días de la semana. Se logró el cegamiento al no decir qué matrona sería observada. De los seguimientos del embarazo, se evaluaron los que duraron al menos tres horas. Para garantizar la objetividad, el investigador observó a una matrona durante un máximo de dos días consecutivos y continuó con otra matrona diferente al tercer día. Este proceso continuó hasta que una matrona hizo el seguimiento de cinco mujeres embarazadas, y cada matrona fue observada al menos una vez cuando hacía el seguimiento de una mujer embarazada mientras daba a luz. El investigador informó a las mujeres embarazadas que la matrona que las seguía y cuidaba sería observada como parte del estudio, y que ninguna información perteneciente a ellas sería utilizada fuera del estudio. El diagrama de flujo de recolección de datos se muestra en la Figura 1. Las matronas que fueron incluidas en la prueba piloto no fueron incluidas en la muestra.

**Figura 1:** Diagrama de flujo de la recogida de datos – Sakarya, Turquía, 2019.

### Análisis de los Datos

En el análisis de los datos obtenidos del estudio, la información descriptiva sobre las matronas se proporcionó como distribuciones numéricas. Los resultados obtenidos del Formulario para evaluar las áreas de competencia de las matronas en tecnología del parto y las puntuaciones correspondientes a la evaluación de las áreas de competencia de las matronas se proporcionaron en forma de tabla. Se otorgó un punto a cada aplicación observada durante la evaluación si se cumplía la aplicación. Por lo tanto, la puntuación máxima que se podía obtener de todas las aplicaciones era de 165 puntos si se cumplían todas. Las aplicaciones indicadas en el Formulario de Evaluación de la Tecnología Utilizada en el Parto según el área de competencia y la categoría en la que se evaluó la tecnología utilizada por las matronas durante el parto, y las observaciones de cada matrona que siguió a las mujeres embarazadas se puntuaron y se incluyeron en la tabla de Excel. Las matronas fueron evaluadas en función de las aplicaciones que cumplieron para cinco mujeres embarazadas.

## RESULTADOS

Los resultados del presente estudio se presentaron bajo los dos epígrafes siguientes: las áreas de competencia de las matronas en la tecnología del parto y la tecnología utilizada en el parto (Tabla 2).

**Tabla 2:** Resultados relativos a las áreas de competencia de las matronas en la tecnología del parto – Sakarya, Turquía, 2019.

Dominio	Subdominios (puntuaciones)	Número de solicitudes en cada subdominio	Matronas														
			M1	M2	M3	M4	M5	M6	M7	M8	M9	M10	M11	M12	M13	M14	Media
Habilidades interpersonales	Atención (20 puntos)	4	7	6	3	5	5	0	5	6	5	5	6	8	8	6	7.5
	Comunicación (20 puntos)	4	15	17	16	14	14	13	14	17	13	17	12	16	14	14	15
	Atención centrada en la mujer (10 puntos)	2	8	4	5	5	7	6	6	10	7	9	8	8	9	7	7.2
Apoyar el conocimiento teórico	Conocimientos de obstetricia (20 puntos)	4	14	2	20	14	20	20	20	20	18	20	20	20	20	20	18
Conocimientos profesionales	Responsabilidad profesional y responsabilidad de la matrona (30 puntos)	6	29	21	27	21	30	30	30	30	28	30	30	30	30	30	28
	Habilidades para utilizar dispositivos (30 puntos)	6	30	30	26	30	24	24	30	30	30	30	30	30	30	30	29.3
Competencia clínica	Toma de decisiones (20 puntos)	4	16	9	16	15	16	15	15	16	15	15	15	16	15	15	15.2
	Habilidades tradicionales de partería** (15 puntos)	3	4 (4V)	4 (4V)	4(3V-1F)	3 (2V-1F)	6 (4V-2F)	4 (1V- 3F)	8 (5V- 3F)	5 (4V- 1F)	6 (4V-2F)	10 (5V- 5F)	8 (1A- 4V- 3F)	11(2A- 5V- 4F)	10 (1A- 5V- 4F)	10 (1A- 5V- 4F)	6.9
Total		33	123	93	117	107	122	112	128	134	122	136	129	139	136	132	123.6

*M = Matrona

** E: Examen vaginal, F: Escuchar el ritmo cardíaco fetal con un fetoscopio, A: Examen abdominal.

Durante la observación, se registró cuántas veces una matrona cumplió con las solicitudes durante el seguimiento de cinco mujeres embarazadas. Debido a que se otorgaba un punto a cada solicitud observada durante la evaluación si la solicitud se cumplía, la puntuación máxima que una matrona podía obtener de todas las solicitudes era de 165 puntos si cumplía con todas las solicitudes. Sin embargo, las puntuaciones obtenidas por las matronas variaron entre 93 y 139 puntos, que estaban por debajo de la puntuación máxima posible, como se ve en la Tabla 2. Especialmente las puntuaciones obtenidas de los subdominios «cuidado», «cuidado centrado en la mujer» y «habilidades tradicionales de partería» por todas las matronas estaban por debajo de la media. *2-Los resultados de la evaluación de la tecnología utilizada por las matronas en el parto según sus áreas de competencia en tecnología del parto se muestran en la Tabla 3. Durante la observación, las matronas fueron evaluadas en 3 dominios, 5 subdominios y 18 aplicaciones*

**Tabla 3:** Resultados de la evaluación de la tecnología utilizada por las matronas en el parto según sus áreas de competencia en tecnología del parto – Sakarya, Turquía, 2019.

Dominio	Subdominios	Categoría	Aplicaciones	Matronas														
				Nivel tecnológico	1	2	3	4	5	6	7	8	9	10	11	12	13	14
Interpersonales	Atención centrada en la mujer	Control del trabajo	Alimentación oral	Cuidados de partería de apoyo	3	0	3	1	3	1	2	4	1	2	2	4	1	1
			Hidratación intravenosa		3	1	1	3	1	2	1	2	1	2	3	3	0	2
	Apoyar el conocimiento teórico	Conocimientos de obstetricia	Programa de reanimación neonatal (PRN)	Alta tecnología	1	1	2	1	0	1	1	0	1	3	1	0	0	1
		Control del trabajo	Amniotomía	Baja tecnología	1	1	0	0	1	0	0	0	2	1	0	0	2	0
			Dosiflow (para inducción)		1	1	2	3	2	2	3	2	0	5	2	2	1	1
			Prostaglandina		1	1	0	0	0	1	0	1	0	1	0	1	1	0
			Profilaxis antibiótica	partería de apoyo	3	1	1	0	0	0	2	0	0	1	0	0	0	1
			Cateterismo BLADDER		0	0	2	1	2	1	1	0	1	2	1	0	1	2
			episiotomía		1	0	1	1	1	1	0	1	0	1	2	1	1	3
Conocimientos profesionales	Responsabilidad profesional y responsabilidad de las matronas	Burocracia	Grabación por ordenador	Alta tecnología	5	5	5	5	5	5	5	5	5	5	5	5	5	5
		Seguimiento de la madre y el feto	Uso de un partograma	Cuidados de partería de apoyo	4	1	1	1	1	0	0	1	2	0	1	2	0	2
clínica	Habilidades para utilizar dispositivos	Seguimiento de la madre y el feto	Control electrónico de las constantes vitales	Alta	0	0	0	0	1	0	0	0	0	0	0	0	2	0
			Monitorización con cardiotocógrafo (CTG)		4	5	4	5	4	4	5	5	5	5	5	5	5	5
		Control del trabajo	Inducción con bomba de infusión electrónica		2	1	0	0	0	0	0	0	1	0	0	0	0	0
		Seguimiento de la madre y el feto	Control del pulso y la tensión arterial Manualmente	Baja tecnología	1	2	0	2	1	0	1	3	3	1	0	0	4	0
	Conocimientos tradicionales de partería	Seguimiento de la madre y el feto	Doppler manual (fetoscopio)	Baja tecnología	1	0	0	1	1	2	1	1	2	3	2	4	3	3
		Alivio del dolor	Pilates	Cuidados de partería de apoyo	0	0	0	0	0	0	0	0	1	0	0	0	2	0
			Masaje			0	0	0	0	0	0	0	0	0	0	0	0	0

La tecnología utilizada por las matronas en el parto según sus áreas de competencia en tecnología del parto se muestra en la Tabla 3. Se registró cada vez que una matrona cumplió con estas aplicaciones durante cinco seguimientos. Mientras que todas las matronas implementaron el monitoreo CTG de alta tecnología y el registro informático en todas las mujeres embarazadas a las que dieron seguimiento, 10 matronas implementaron el NRP para el cuidado del recién nacido, pero solo en una de cada cinco mujeres embarazadas. Lograron aplicaciones de baja tecnología en un pequeño número de casos. En el subdominio de atención obstétrica de apoyo, solo una matrona implementó aplicaciones de pilates (dos matronas) y masajes, 13 monitorearon la inducción con dosiflow, 13 aplicaron hidrataciones intravenosas por infusión, 12 utilizaron Doppler manual, 10 utilizaron un partograma para el seguimiento de la madre y el feto, 10 realizaron cateterismo vesical, 11 realizaron episiotomía y 9 monitorearon el pulso y la presión arterial manualmente.

## DISCUSIÓN

En el presente estudio, se evaluaron mediante observación y entrevistas las competencias relacionadas con el uso de la tecnología del parto de las matronas que trabajan en la clínica de obstetricia. El estudio se llevó a cabo utilizando el modelo de competencias en tecnología del parto. Debido al número limitado de estudios en los que se utilizó este modelo, la comparación de los resultados se realizó utilizando otra literatura relacionada con el tema.

La competencia en la tecnología utilizada en el parto también asegura el vínculo entre la teoría y la práctica. Entre las áreas de competencia de las matronas que trabajan en la sala de partos se encuentran el monitoreo y el cuidado de la mujer embarazada a la que siguen. En el proceso que comienza con el ingreso de la mujer embarazada a la clínica, se debe tomar su anamnesis, se le debe realizar un examen físico y vaginal, y se debe hacer su evaluación de riesgos. Las matronas también deben comunicarse eficazmente con la embarazada de acuerdo con sus responsabilidades y competencias profesionales, deben preocuparse por la embarazada tomando decisiones sobre su cuidado y deben ser capaces de utilizar los dispositivos que se pueden utilizar en el seguimiento del parto e informarla sobre la finalidad de los dispositivos utilizados. Las matronas también deben proteger la privacidad de la embarazada siendo respetuosas con la madre y su familia durante todas las prácticas, deben tener en cuenta los deseos de la embarazada mientras la atienden, deben proporcionar apoyo emocional y psicológico, y deben informar a la embarazada sobre todas las aplicaciones durante y después de que se someta a ellas. Las matronas deben registrar los resultados que obtengan en el Formulario de Observación de la Matrona después del examen y la evaluación^(3, 10)^. Las puntuaciones que las matronas participantes obtuvieron en el formulario de observación utilizado para evaluar las competencias de las matronas en la tecnología del parto variaron entre 93 y 139. De las matronas, la que obtuvo la puntuación más baja llevaba seis meses trabajando como matrona en la clínica de obstetricia. La que obtuvo la puntuación más alta era una matrona experimentada que llevaba 30 años trabajando en la sala de partos. Del mismo modo, otras matronas que habían trabajado en la sala de partos durante muchos años obtuvieron puntuaciones altas. Estos resultados revelaron la importancia de la experiencia para adquirir competencia en la tecnología del parto. El análisis de las áreas de competencia en términos de categorías demostró que las puntuaciones más bajas se obtuvieron en el dominio de las habilidades interpersonales. Mientras que las puntuaciones obtenidas en los dominios de «Conocimientos teóricos de apoyo» y «Conocimientos profesionales» fueron altas, las puntuaciones obtenidas en el dominio de «Competencia clínica» no lo fueron.

El dominio de habilidades interpersonales incluye los subdominios de cuidado, comunicación y atención centrada en la mujer. Las habilidades interpersonales son muy importantes tanto en el cuidado de la mujer embarazada como en las relaciones de equipo durante el parto. Las habilidades interpersonales también se incluyen en los códigos éticos de partería de la ICM^(4)^. Sin embargo, las puntuaciones que obtuvieron en los subdominios de «cuidado» y «atención centrada en la mujer» fueron inferiores a las que obtuvieron en el subdominio de comunicación. En sus Directrices sobre la atención durante el parto, la OMS (2018) recomienda que se tengan en cuenta las declaraciones y necesidades de las mujeres en el ámbito de la atención respetuosa de la maternidad. La atención a las mujeres se refiere al respeto tanto de los derechos individuales de la mujer como de la atención individualizada. Comienza por situar a la mujer en el centro de la atención, y la «atención centrada en la mujer» debe ser la filosofía básica en el parto. El centro de la atención debe ser la «mujer/madre». Aunque las puntuaciones que las matronas obtuvieron en el subdominio de «atención centrada en la mujer» estaban por encima de la media, no estaban al nivel deseado. Sin embargo, las matronas que participaron en la investigación obtuvieron altas puntuaciones en el subdominio de «comunicación». El «cuidado» junto con las «habilidades de comunicación» desempeñan un papel en el establecimiento de una interacción y cooperación más eficaces entre las matronas y las mujeres embarazadas, las madres y el personal sanitario^(12)^. Además, el uso eficaz de las habilidades de comunicación al tomar la anamnesis y tener en cuenta las declaraciones de la madre garantizará la detección de los riesgos en una fase temprana, y así se hará un seguimiento saludable de la mujer embarazada y se aliviarán sus preocupaciones^(13, 14)^. Por lo tanto, si las matronas van a proporcionar apoyo continuo a las mujeres embarazadas durante el parto, deben tener buenas habilidades de comunicación y proporcionar una atención respetuosa a las mujeres embarazadas. El establecimiento de una comunicación eficaz por parte de las matronas es el factor principal para la individualidad del parto y garantizará la participación activa de la embarazada en el proceso y la animará durante el parto. Así, se reduce el uso de analgesia y anestesia, se reduce la tasa de intervenciones, aumenta la confianza en sí misma y la sensación de éxito de la embarazada, se asegura su participación activa en el parto, se acorta la duración del mismo, se crea un entorno de parto seguro con el apoyo continuo de las matronas, se fortalece el vínculo madre-hijo, se garantiza una lactancia eficaz, se facilita la adaptación al posparto y aumenta la comodidad de la embarazada y la satisfacción materna en el parto^(12, 13, 15, 16, 17, 18, 19)^. Se observó que las matronas durante el horario laboral diurno no podían dedicar suficiente tiempo a las mujeres embarazadas, probablemente porque el nivel de trabajo burocrático durante el horario laboral diurno en la clínica era alto, el número de miembros del equipo que trabajaban en la clínica era elevado y cada miembro del equipo tenía ciertas expectativas de las matronas.

En el presente estudio, se observó que todas las matronas llevaron a cabo la categoría de «burocracia» en el subdominio de «conocimientos profesionales» para todas las mujeres embarazadas. En esta categoría, las matronas mostraron los elementos de la competencia burocrática en las aplicaciones. Entre las razones por las que la burocracia es más dominante se encuentran las preocupaciones por los litigios legales, la importancia que se da a los protocolos y directrices, y la inclusión de los médicos en la responsabilidad^(3)^. El análisis de los resultados demostró que todas las matronas realizaron la aplicación de «registro informático» en la categoría de «burocracia». Esto demuestra que, aunque las matronas eran conscientes de sus responsabilidades y obligaciones profesionales, también daban importancia al registro, lo que indica que trabajaban como «secretarias médicas».

La inadecuación de las matronas observada en el comportamiento de atención a las mujeres se debió probablemente a las diferencias entre sus edades, años de servicio en la profesión y en la sala de partos, y niveles de educación. Otros dos dominios de competencia en tecnología del parto son *Soporte de Conocimientos Teóricos* (conocimientos de partería) y *Conocimientos Profesionales* (responsabilidad profesional y responsabilidad de la matrona). Las puntuaciones obtenidas por las matronas en estos dominios de competencia en tecnología del parto fueron altas.

Las matronas llevaron a cabo la aplicación de «cuidados del recién nacido» en el subdominio «Conocimientos de partería» utilizando alta tecnología. En el presente estudio, se observó que las matronas implementaron las aplicaciones en la segunda fase del parto al menos una vez, pero desafortunadamente, no todas las matronas. De las 14 matronas, diez utilizaron alta tecnología mientras realizaban cuidados al recién nacido porque habían utilizado previamente esa tecnología al menos una vez. Las aplicaciones como la amniotomía, la inducción con dosiflow y la prostaglandina, que se encuentran en la categoría «Control del parto» del subdominio de conocimientos obstétricos, se realizaron utilizando baja tecnología. Como se ve en la Tabla 3, las matronas no cumplieron con las aplicaciones como el uso de antibióticos, el cateterismo vesical y la episiotomía incluidas en la categoría Control del parto en todas las mujeres embarazadas. La aplicación de estas aplicaciones a un ritmo bajo se considera un enfoque positivo porque el conocimiento de partería mencionado aquí significa intervención en el parto. Debido a que el hospital donde se realizó el presente estudio es de tercer nivel, las decisiones sobre la aplicación de la inducción y la profilaxis con antibióticos las toman los médicos. Las matronas solo son responsables de realizar las aplicaciones y seguir el proceso. Además, deben informar a los médicos sobre las complicaciones que surjan o puedan surgir. En su estudio realizado con mujeres embarazadas primíparas, Balcik Colak y Ozturk Can^(19, 20)^ afirmaron que el 88% de las mujeres embarazadas se sometieron a un cateterismo vesical durante el parto, lo que coincide con nuestros hallazgos. Dado que las intervenciones en el parto se realizan con mucha frecuencia en todo el mundo, la OMS no recomienda la intervención en el parto a menos que sea necesaria^(12)^.

En el dominio de Competencia Clínica, todas las matronas obtuvieron puntuaciones altas en el subdominio «Habilidades para usar dispositivos». Sin embargo, la puntuación que obtuvieron en el subdominio «Habilidades tradicionales de partería» estuvo por debajo de la media. Además, se observó que solo una matrona realizó masajes. Aunque 12 matronas monitorearon la FCF con un fetoscopio, que está en la misma categoría, no lo llevaron a cabo en todas las mujeres embarazadas a las que dieron seguimiento. En su lugar, prefirieron el CTG. La OMS no recomienda el examen vaginal continuo durante el parto. En el presente estudio, la razón por la que las matronas no realizaron un examen abdominal adecuado fue probablemente porque los médicos eran los que decidían los procedimientos a los que debían someterse las mujeres embarazadas. Por eso realizaron CTG y USG.

Además, las matronas no monitorearon los signos vitales electrónicamente en la categoría de monitoreo de la madre y el feto, pero todas las matronas utilizaron CTG. Estas dos aplicaciones se llevan a cabo utilizando tecnología avanzada. Sin embargo, esto indica que las matronas no monitorearon las contracciones tocando a la mujer embarazada con sus manos. Dado que el seguimiento de la embarazada con CTG también permitía realizar evaluaciones en menos tiempo, las matronas no mantenían un contacto prolongado con la embarazada. Como las matronas pasan poco tiempo con la embarazada, no pueden escucharla adecuadamente y, por lo tanto, no pueden realizar las intervenciones adecuadas para aliviar su dolor. Como práctica de rutina, el CTG es importante ya que se utiliza para determinar y detectar riesgos fetales de forma temprana y para prevenir complicaciones^(21)^. La OMS, el Colegio Americano de Enfermeras Matronas y el Instituto Nacional para la Excelencia en Salud y Atención (NICE) en el Reino Unido, todos los cuales se ocupan de la salud maternoinfantil, no recomiendan el uso rutinario del CTG, pero sí recomiendan la auscultación intermitente para monitorear al feto, excepto en situaciones de riesgo^(12, 22, 23)^. En algunos estudios, en los que se incluyeron las opiniones de los profesionales sanitarios sobre la monitorización fetal en el parto, se informó de que los profesionales sanitarios se sentían más seguros con la CTG y querían utilizarla debido a preocupaciones legales^(24)^.

Entre las aplicaciones tecnológicas utilizadas en el seguimiento del trabajo de parto, el monitoreo electrónico de los signos vitales y la inducción con una bomba de infusión electrónica se consideran aplicaciones de alta tecnología, mientras que el monitoreo manual de los signos vitales se considera una aplicación de baja tecnología^(3)^. Solo dos de las matronas que participaron en el presente estudio monitorearon los signos vitales electrónicamente, porque las otras matronas declararon que no confiaban en las mediciones digitales para medir los signos vitales. Por lo tanto, la mayoría de las matronas utilizaron el sistema manual de baja tecnología para monitorear la frecuencia cardíaca y la presión arterial.

Solo tres de ellas realizaron aplicaciones de inducción con bomba de infusión electrónica. Utilizaron activamente la baja tecnología en aplicaciones de inducción porque tenían problemas para usar la bomba de inducción y carecían de conocimientos sobre cómo usarla, lo que sugiere que necesitan capacitación sobre el uso de dispositivos y tecnología.

Se ha informado de que la evaluación de la cabeza fetal con ecografía de rutina no es beneficiosa en embarazos de bajo riesgo y que está asociada con mayores tasas de cesáreas^(25)^. En algunos estudios observacionales, se ha informado de que la ecografía repetitiva da mejores resultados que el examen clínico manual en el diagnóstico del parto que no progresa en la primera y segunda etapa del parto^(26)^. El uso de métodos tecnológicos como la CTG y la USG tiene un papel muy importante en la determinación de situaciones de riesgo. Sin embargo, su uso rutinario provoca una disminución en el contacto con la mujer embarazada y reduce la interacción con ella. Se ha informado de que el contacto tiene efectos fisiológicos y psicológicos positivos para aliviar el dolor del parto, reducir significativamente los niveles de ansiedad y dolor y acortar la duración del parto^(27)^. Además, si las matronas conocen sus áreas de competencia y autonomía, podrán tomar la decisión correcta sobre el seguimiento de las mujeres embarazadas ingresadas en la clínica de obstetricia, proporcionar la información y orientación necesarias y garantizar el máximo beneficio para la salud de la madre y el bebé.

Dado que el uso de dispositivos tecnológicos sirve al propósito principal, el uso de la tecnología en el parto se convierte en la principal necesidad. Cabe señalar que el uso de dispositivos no es un objetivo, sino una herramienta, y debe aumentarse la conciencia sobre su uso previsto^(30)^. En sus estudios, Cowie y Floyd^(29)^ expresaron su preocupación por el hecho de que las matronas estuvieran poniendo fin a las funciones reales de la partería debido a los dispositivos tecnológicos que utilizaban en las prácticas de partería. En consonancia con los resultados del presente estudio, los datos obtenidos durante las entrevistas mantenidas con las matronas que participaron en el estudio de Cowie y Floyd indicaron que el uso de la tecnología disminuía el contacto con la embarazada y que las habilidades y prácticas manuales en la partería pasaban a un segundo plano.

Las puntuaciones que las matronas participantes obtuvieron en el subdominio «Toma de decisiones» del dominio «Competencia clínica» estaban por encima de la media, pero no a un nivel alto. En el presente estudio, los niveles de toma de decisiones clínicas de las participantes no se evaluaron por separado. Sin embargo, se cree que el poder de decisión y la autonomía de las matronas deberían reforzarse aún más. Esto también promoverá la atención centrada en la mujer^(19)^. Se cree que la matrona que obtuvo la puntuación más baja en el subdominio «Toma de decisiones» aún no ha adquirido competencia en la toma de decisiones porque su tiempo de servicio en la sala de partos fue corto y porque su formación en partería fue a nivel de escuela secundaria.

El enfoque de las matronas debe basarse en la atención integral. Debe centrarse en el uso individualizado de la tecnología. El uso rutinario de la tecnología debe limitarse, porque conduce a una disminución en la prestación de atención individualizada y afecta negativamente el proceso de estar con las mujeres. Es importante analizar los efectos del uso de la tecnología en la evaluación del seguimiento intraparto y los resultados sobre el papel de las matronas y sobre la experiencia de parto de la mujer^(30)^. En el modelo tecnocrático, se piensa que se facilita el trabajo de los profesionales de la salud^(2, 6)^. Según los resultados del presente estudio, las matronas defendieron nuevas tipologías de competencias profesionales en la fase de entrevistas, pero aplicaron la tipología de competencias burocráticas. Según los resultados de las entrevistas realizadas a las matronas en el estudio de Crozier et al.^(3)^, aunque defendían la competencia profesional, mostraban en la práctica las características de la competencia burocrática.

El hecho de que los niveles de educación y la duración del servicio en la profesión de las matronas participantes variaran y que algunas de ellas recibieran formación distinta a la de matrona puede haber afectado a su competencia. Además, las condiciones físicas del área de parto, las condiciones de trabajo centradas en el trabajo, el entorno inadecuado del área de parto, un número inadecuado de personal y una formación en el servicio inadecuada también pueden haber afectado a los resultados del presente estudio. Sin embargo, estas reflexiones solo deben considerarse como suposiciones, ya que el objetivo principal del presente estudio no era determinar los factores que afectaban a las competencias relacionadas con las tecnologías del parto. Además del uso de la tecnología, también deben incluirse las prácticas tradicionales de partería, como estar con la paciente, escucharla, asesorarla y mostrar empatía, para no alejarse de la atención centrada en la matrona.

## CONCLUSIÓN

En el presente estudio observacional realizado para investigar las áreas de competencia de las matronas y el uso de la tecnología durante el parto, se observó que las matronas participantes tenían algunas deficiencias en cuanto al uso de la tecnología en el parto y en áreas como las habilidades interpersonales y la competencia clínica. Los resultados obtenidos en base a nuestras observaciones son los siguientes:

- En general, las matronas participantes que trabajaban en la sala de partos utilizaban dispositivos de baja tecnología.
- Las habilidades tradicionales de partería utilizadas por las matronas participantes durante el parto eran inadecuadas.

De acuerdo con estos resultados, para mejorar las áreas de competencia de las matronas y eliminar sus deficiencias en el uso de la tecnología durante el parto, se recomienda que:

- En la formación de matronas, se les imparta educación tecnológica sobre cómo utilizar los dispositivos tecnológicos y cómo interpretar e intervenir correctamente en los resultados de registro de los dispositivos, y que sus necesidades de competencia tecnológica se satisfagan, apoyen y mejoren con formación.
- Al proporcionar asesoramiento, las matronas deben informar a las mujeres sobre el uso de la tecnología para reducir sus efectos negativos en la salud de las mujeres embarazadas, las madres y los bebés, que deben asegurarse de que se tomen las precauciones necesarias en el uso de los dispositivos y que deben cumplir su función de defensa en la formación y el asesoramiento.
- Los responsables políticos deben actualizar los datos sobre el uso de la tecnología en los programas de formación de matronas y ampliar la autoridad de las matronas en lo que respecta al uso de dispositivos tecnológicos.
